# *catena*-Poly[cobalt(II)-di-μ-chlorido-κ^4^ *Cl*:*Cl*-μ-1,5-dimethyl-1*H*-tetra­zole-κ^2^ *N* ^3^:*N* ^4^]: an X-ray powder investigation

**DOI:** 10.1107/S1600536809003018

**Published:** 2009-01-28

**Authors:** Ludmila S. Ivashkevich, Alexander S. Lyakhov, Anastasiya P. Mosalkova, Pavel N. Gaponik, Oleg A. Ivashkevich

**Affiliations:** a Research Institute for Physico-Chemical Problems of Belarusian State University, Leningradskaya Street 14, Minsk 220030, Belarus

## Abstract

The asymmetric unit of the title compound, [CoCl_2_(C_3_H_6_N_4_)]_*n*_, contains two Co atoms, both lying on inversion centres, two Cl atoms and one 1,5-dimethyl­tetra­zole ligand. The coordination polyhedra of both Co atoms adopt flattened octa­hedral geometry, with two N atoms from two ligands in axial positions and four Cl atoms in equatorial sites. Neighbouring Co atoms are linked together *via* two bridging Cl atoms and one tetra­zole ring to form polymeric chains running along the *a* axis.

## Related literature

For the crystal structure of a related Cu complex, see: Ivashkevich *et al.* (2006 ▶). For values of radii for ions with octahedral coordination and mol­ecular geometric parameters, see: Shannon (1976 ▶) and Allen (2002 ▶), respectively. For details of the indexing algorithm, see: Werner *et al.* (1985 ▶).
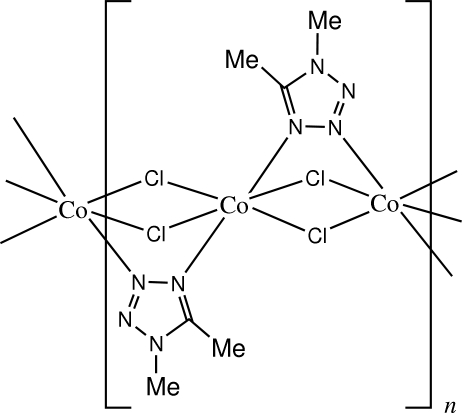


## Experimental

### 

#### Crystal data

[CoCl_2_(C_3_H_6_N_4_)]
*M* *_r_* = 227.95
Triclinic, 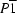

*a* = 6.7159 (4) Å
*b* = 7.5986 (4) Å
*c* = 8.9231 (5) Å
α = 108.639 (2)°
β = 107.259 (3)°
γ = 105.769 (3)°
*V* = 376.72 (4) Å^3^
*Z* = 2
Co *K*α radiation
*T* = 295 K
Specimen shape: flat sheet
30 × 30 × 1 mm
Specimen prepared at 100 kPa
Specimen prepared at 295 K
Particle morphology: finely ground powder, light-violet

#### Data collection

HZG-4A (Carl Zeiss, Jena) diffractometer
Specimen mounting: packed powder pellet
Specimen mounted in reflection mode
Scan method: step
2θ_min_ = 11.0, 2θ_max_ = 130.0°
Increment in 2θ = 0.02°

#### Refinement

*R*_p_ = 0.018
*R*_wp_ = 0.024
*R*_exp_ = 0.025
*R*_B_ = 0.023
*S* = 0.97
Wavelength of incident radiation: 1.79021 Å
Excluded region(s): none
Profile function: psevdo-Voigt, η = 0.664(5)
896 reflections
45 parameters
7 restraints
H-atom parameters constrained
Preferred orientation correction: none

### 

Data collection: local program; cell refinement: *FULLPROF* (Rodríguez-Carvajal, 2001 ▶); data reduction: local program; program(s) used to refine structure: *FULLPROF*; molecular graphics: *PLATON* (Spek, 2003 ▶); software used to prepare material for publication: *FULLPROF*, *SHELXL97* (Sheldrick, 2008 ▶) and *PLATON* (Spek, 2003 ▶).

## Supplementary Material

Crystal structure: contains datablocks global, I. DOI: 10.1107/S1600536809003018/cv2491sup1.cif

Rietveld powder data: contains datablocks I. DOI: 10.1107/S1600536809003018/cv2491Isup2.rtv

Additional supplementary materials:  crystallographic information; 3D view; checkCIF report

## Figures and Tables

**Table 1:** Selected bond lengths (Å)

Co1—Cl2	2.461 (3)
Co1—N4	2.224 (10)
Co1—Cl1^i^	2.482 (3)
Co2—Cl1	2.446 (3)
Co2—N3	2.111 (9)
Co2—Cl2^ii^	2.479 (3)

Symmetry codes: (i) 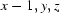; (ii) 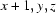.

**Fig. 1.**
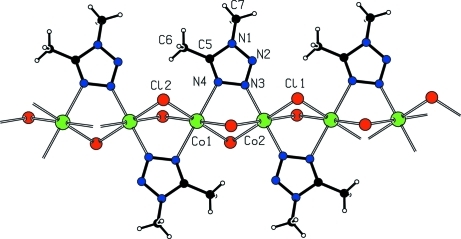
A portion of the polymeric chain of (I) running along the a axis. The atomic numbering scheme is given for the asymmetric unit.

**Fig. 2.**
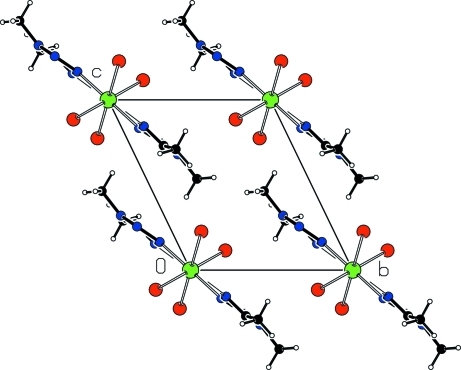
The crystal structure of (I), viewed along the a axis.

**Fig. 3.**
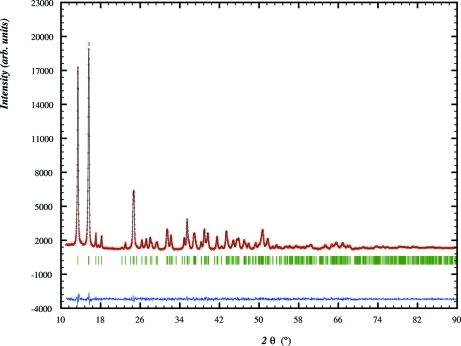
The Rietveld plot for (I), showing the observed (red circles), calculated (black line) and difference (blue line) patterns. The reflection positions are shown as vertical bars above the difference pattern.

## supplementary crystallographic information

### Comment

In our previous paper (Ivashkevich *et al.*, 2006) we reported the crystal structure of copper(II) chloride complex with 1,5-dimethyltetrazole, CuCl_2_L. That was the first experimental findings of bridge coordination of 1,5-disubstituted tetrazoles through the tetrazole ring bridge N3—N4. In the present work, we report another example of such type complexes, namely the title complex of cobalt(II) chloride with 1,5-dimethyltetrazole, (I).

Complex (I) has a 1:1 metal-to-ligand ratio of cobalt(II) with the 1,5-dimethyltetrazole. The asymmetric unit contains two Co atoms, both lying on inversion centres, two Cl atoms and one 1,5-dimethyltetrazole molecule, all in general positions. Co1 is bonded to the tetrazole ring atoms N4, whereas Co2 coordinates the tetrazole ring atoms N3 (Fig. 1). The tetrazole ring geometry is typical of 1- and 1,5-substituted tetrazoles. The complex is a one-dimensional coordination polymer, with polymeric chains running along the *a* axis (Fig.1,2). The chains are formed due to chloride bridges and the tetrazole ring bridges N3—N4 between the neighbouring Co atoms.

Complex (I) is isotypic with the above copper(II) analogue, and it is of interest to compare the structures of the compounds. Whereas Cu coordination octahedra show essential elongation of axial Cu—Cl bonds compared to equatorial Cu—Cl and Cu—N ones, Co octahedra are flattened, with axial Co—N bonds and very similar in lengths equatorial Co—Cl bonds. In Co1 and Co2 octahedra, the difference between the axial and equatorial bond lengths (Table 1), being much less than that in the Cu octahedra, may be related to difference in size of Cl and N atoms. However, essential elongation of the Cu octahedra is probably induced by the Jahn-Teller effect. In complex (I), closely spaced values of all Co—Cl bond lengths are responsible for rather symmetrical chloride bridges between the neighbouring Co atoms in polymeric chains, in contrast to copper(II) analogue with non-symmetrical chloride bridges.

Comparing the cell volumes of the two isotypic compounds [374.15 (4) Å^3^ for Cu complex and 376.72 (4) Å^3^ for Co one and taking into account octahedral ionic radii (Shannon, 1976) of Cu^II^ (0.73 Å) and Co^II^ (0.65 Å for low-spin state and 0.75 Å for high-spin state), one may expect that cations Co^II^ in complex(I) are in high-spin state at room temperature. This conclusion is confirmed by EPR investigation of the complex, which does not reveal EPR spectra at room temperature. As known, Co^II^ cations in high-spin state shows EPR signals only at very low temperatures.

### Experimental

A solution, containing 1.17 g (0.005 mol) of CoCl_2_^.^6H_2_O in 10 ml of a mixture of methanol and triethyl orthoformate (*v*/*v* =1:1), was added to a solution of 1,5-dimethyltetrazole (0.5 g, 0.0051 mol) in 10 ml of the same solvent mixture. After stirring the reaction mixture at 323–333 K C for 0.5 h, the obtained light-violet crystals of (I) were filtered off, washed with methanol (2 × 5 ml), and dried in air [0.96 g, yield 84%; m.p. 633 K (decomposition)]. Calc. (%): Co 25.8, Cl 30.6. Found (%): Co 25.7, Cl 31.1. IR (cm^-1^): 3044 (*m*), 3025 (*s*), 2962 (*m*), 2938 (*m*), 1535 (*s*), 1480 (*m*), 1454 (w), 1409 (w), 1396 (*m*), 1383 (*s*), 1332 (*s*), 1265 (*m*), 1235 (*s*), 1144 (*s*), 1090 (w), 1060 (*s*), 1031 (*m*), 739 (*s*), 681 (*m*), 607 (w), 565 (w), 525 (w).

### Refinement

For complex (I), a triclinic unit cell (a = 6.728, b = 7.596, c = 8.764 Å, α = 109.62, β = 102.96, γ = 105.70°) was determined using the indexing program TREOR90 (Werner *et al.*, 1985). The obtained values as well as observed resemblance of powder patterns indicated isotypism of (I) with investigated earlier coordination polymers CuCl_2_L, where *L* = 1,5-dimethyltetrazole, crystallizing in the space group *P*1 (Ivashkevich *et al.*, 2006). This space group and the atomic coordinates of the above copper(II) complex were used as starting parameters for the Rietveld refinement of (I) with the FULLPROF program (Rodríguez-Carvajal, 2001). However, the refinement found difficulty in reaching the agreement of experimental and calculated intensities of reflections. From this fact an assumption was made that the above unit-cell dimensions were inconsistent with the initial atomic coordinates. Search for alternative unit cells resulted in the following cell: a' = 6.728, b' = 7.596, c' = 8.997 Å; α' = 108.35, β' = 107.50, γ' = 105.70°, which is of the same volume and related to the first one by the vector transformation a' =-a, b' =-b, c' = a + b + c. This cell provided a good agreement of the observed and calculated intensities.

Background intensity was found by Fourier filtering technique as implemented in the FULLPROF program, under visual inspection of the resulting background curve. Correction for profile asymmetry was made for reflections up to 2θ=25°.

The H atoms of the methyl groups were placed in geometrically calculated positions using the program *SHELXL97* (Sheldrick, 2008), with displacement parameter B_iso_(H)=1.5B_iso_(C). Non-H atoms were refined isotropically. Four independent B_iso_ parameters were employed: one for the two Co, one for the two Cl atoms, one for all N and C atoms of the tetrazole ring, and one for the C atoms of the methyl groups.

For the refinement, suitable restraints were imposed on bond lengths of the ligand molecule, based on a geometry analysis of 1,5-alkyltetrazoles (Cambridge Structural Database, version 5.29 of November 2007; Allen, 2002). The restraints were set as d(3σ), where d are mean values of bond distances resulting from a CSD survey, and σ are their s.u. values. For the refined atomic coordinates of (I), the s.u. values are taken from the software and likely to be underestimated.

The observed, calculated and difference diffraction patterns for the refined crystal structure are shown in Fig. 3 (2θ range of 11–90 ° is presented).

### Crystal data

**Table:**

[CoCl_2_(C_3_H_6_N_4_)]	*V* = 376.72 (4) Å^3^
*M**_r_* = 227.95	*Z* = 2
Triclinic, *P*1	*F*(000) = 226
Hall symbol: -P 1	*D*_x_ = 2.010 Mg m^−^^3^
*a* = 6.7159 (4) Å	Co *K*α radiation, λ = 1.79021 Å
*b* = 7.5986 (4) Å	*T* = 295 K
*c* = 8.9231 (5) Å	Particle morphology: finely ground powder
α = 108.639 (2)°	light-violet
β = 107.259 (3)°	flat sheet, 30 × 30 mm
γ = 105.769 (3)°	Specimen preparation: Prepared at 295 K and 100 kPa

### Data collection

**Table:**

HZG-4A (Carl Zeiss, Jena) diffractometer	Data collection mode: reflection
Radiation source: fine-focus sealed X-ray tube, BSV-29	Scan method: step
Fe filtered	2θ_min_ = 11.00°, 2θ_max_ = 130.00°, 2θ_step_ = 0.02°
Specimen mounting: packed powder pellet	

### Refinement

**Table:**

Refinement on *I*_net_	Profile function: psevdo-Voigt, η = 0.664(5)
Least-squares matrix: full with fixed elements per cycle	45 parameters
*R*_p_ = 0.018	7 restraints
*R*_wp_ = 0.024	0 constraints
*R*_exp_ = 0.025	H-atom parameters constrained
*R*_Bragg_ = 0.023	Weighting scheme based on measured s.u.'s
χ^2^ = 0.941	(Δ/σ)_max_ = 0.002
5951 data points	Background function: Fourier filtering
Excluded region(s): none	Preferred orientation correction: none

### Special details

**Table:**

Geometry. Bond distances, angles *etc*. have been calculated using the rounded fractional coordinates. All su's are estimated from the variances of the (full) variance-covariance matrix. The cell e.s.d.'s are taken into account in the estimation of distances, angles and torsion angles

### Fractional atomic coordinates and isotropic or equivalent isotropic displacement parameters (Å^2^)

**Table:**

	*x*	*y*	*z*	*U*_iso_*/*U*_eq_	
Co1	0.00000	1.00000	0.00000	0.0183 (8)*	
Co2	0.50000	1.00000	0.00000	0.0183 (8)*	
Cl1	0.6168 (5)	0.7266 (4)	−0.1147 (4)	0.0254 (9)*	
Cl2	−0.1025 (5)	1.1819 (4)	0.2275 (4)	0.0254 (9)*	
N1	0.3124 (10)	0.7423 (14)	0.3267 (10)	0.032 (2)*	
N2	0.4540 (11)	0.8008 (13)	0.2565 (10)	0.032 (2)*	
N3	0.3888 (9)	0.8891 (13)	0.1626 (10)	0.032 (2)*	
N4	0.1745 (10)	0.8607 (14)	0.1504 (11)	0.032 (2)*	
C5	0.1349 (12)	0.783 (2)	0.2552 (15)	0.032 (2)*	
C6	−0.0585 (12)	0.7367 (16)	0.2934 (13)	0.035 (3)*	
H6A	−0.03346	0.68164	0.37682	0.053*	
H6B	−0.19026	0.63786	0.18797	0.053*	
H6C	−0.08251	0.85734	0.33971	0.053*	
C7	0.3895 (17)	0.6847 (16)	0.4623 (10)	0.035 (3)*	
H7A	0.52906	0.66940	0.46948	0.053*	
H7B	0.27627	0.55778	0.43745	0.053*	
H7C	0.41569	0.78730	0.57057	0.053*	

### Geometric parameters (Å, °)

**Table:**

Co1—Cl2	2.461 (3)	N1—C5	1.341 (14)
Co1—N4	2.224 (10)	N1—C7	1.419 (14)
Co1—Cl1^i^	2.482 (3)	N2—N3	1.283 (13)
Co1—Cl2^ii^	2.461 (3)	N3—N4	1.361 (11)
Co1—N4^ii^	2.224 (10)	N4—C5	1.307 (17)
Co1—Cl1^iii^	2.482 (3)	C5—C6	1.421 (15)
Co2—Cl1	2.446 (3)	C6—H6A	0.9600
Co2—N3	2.111 (9)	C6—H6B	0.9600
Co2—Cl2^iv^	2.479 (3)	C6—H6C	0.9600
Co2—Cl2^ii^	2.479 (3)	C7—H7A	0.9600
Co2—Cl1^iii^	2.446 (3)	C7—H7B	0.9600
Co2—N3^iii^	2.111 (9)	C7—H7C	0.9600
N1—N2	1.340 (12)		
			
Cl2—Co1—N4	95.5 (2)	Cl1^iii^—Co2—N3^iii^	94.9 (3)
Cl1^i^—Co1—Cl2	84.91 (11)	Co1^iv^—Cl1—Co2	85.92 (10)
Cl2—Co1—Cl2^ii^	180.00	Co1—Cl2—Co2^i^	85.65 (10)
Cl2—Co1—N4^ii^	84.5 (2)	N2—N1—C5	103.6 (9)
Cl1^iii^—Co1—Cl2	95.09 (11)	N2—N1—C7	118.7 (8)
Cl1^i^—Co1—N4	93.6 (3)	C5—N1—C7	137.0 (10)
Cl2^ii^—Co1—N4	84.5 (2)	N1—N2—N3	113.8 (8)
N4—Co1—N4^ii^	180.00	Co2—N3—N2	136.1 (6)
Cl1^iii^—Co1—N4	86.4 (3)	Co2—N3—N4	119.0 (7)
Cl1^i^—Co1—Cl2^ii^	95.09 (11)	N2—N3—N4	103.5 (8)
Cl1^i^—Co1—N4^ii^	86.4 (3)	Co1—N4—N3	115.1 (7)
Cl1^i^—Co1—Cl1^iii^	180.00	Co1—N4—C5	134.3 (7)
Cl2^ii^—Co1—N4^ii^	95.5 (2)	N3—N4—C5	109.6 (9)
Cl1^iii^—Co1—Cl2^ii^	84.91 (11)	N1—C5—N4	108.8 (9)
Cl1^iii^—Co1—N4^ii^	93.6 (3)	N1—C5—C6	121.2 (12)
Cl1—Co2—N3	94.9 (3)	N4—C5—C6	130.0 (11)
Cl1—Co2—Cl2^iv^	85.31 (11)	C5—C6—H6A	109.00
Cl1—Co2—Cl2^ii^	94.69 (11)	C5—C6—H6B	109.00
Cl1—Co2—Cl1^iii^	180.00	C5—C6—H6C	110.00
Cl1—Co2—N3^iii^	85.1 (3)	H6A—C6—H6B	109.00
Cl2^iv^—Co2—N3	91.3 (2)	H6A—C6—H6C	110.00
Cl2^ii^—Co2—N3	88.7 (2)	H6B—C6—H6C	110.00
Cl1^iii^—Co2—N3	85.1 (3)	N1—C7—H7A	109.00
N3—Co2—N3^iii^	180.00	N1—C7—H7B	109.00
Cl2^iv^—Co2—Cl2^ii^	180.00	N1—C7—H7C	109.00
Cl1^iii^—Co2—Cl2^iv^	94.69 (11)	H7A—C7—H7B	109.00
Cl2^iv^—Co2—N3^iii^	88.7 (2)	H7A—C7—H7C	110.00
Cl1^iii^—Co2—Cl2^ii^	85.31 (11)	H7B—C7—H7C	110.00
Cl2^ii^—Co2—N3^iii^	91.3 (2)		

Symmetry codes: (i) *x*−1, *y*, *z*; (ii) −*x*, −*y*+2, −*z*; (iii) −*x*+1, −*y*+2, −*z*; (iv) *x*+1, *y*, *z*.
